# The combination of mesoglycan and VEGF promotes skin wound repair by enhancing the activation of endothelial cells and fibroblasts and their cross-talk

**DOI:** 10.1038/s41598-022-15227-1

**Published:** 2022-06-30

**Authors:** Raffaella Belvedere, Nunzia Novizio, Silvana Morello, Antonello Petrella

**Affiliations:** Department of Pharmacy, University of Salerno, Via Giovanni Paolo II, 132, 84084 Fisciano, Salerno Italy

**Keywords:** Skin diseases, Cell migration

## Abstract

Skin wound healing requires accurate therapeutic topical managements to accelerate tissue regeneration. Here, for the first time, we found that the association mesoglycan/VEGF has a strong pro-healing activity. In detail, this combination induces angiogenesis in human endothelial cells promoting in turn fibroblasts recruitment. These ones acquire a notable ability to invade the matrigel coating and to secrete an active form of metalloproteinase 2 in presence of endothelial cells treated with mesoglycan/VEGF. Next, by creating intrascapular lesions on the back of C57Bl6 mice, we observed that the topical treatments with the mesoglycan/VEGF promotes the closure of wounds more than the single substances beside the control represented by a saline solution. As revealed by eosin/hematoxylin staining of mice skin biopsies, treatment with the combination mesoglycan/VEGF allows the formation of a well-structured matrix with a significant number of new vessels. Immunofluorescence analyses have revealed the presence of endothelial cells at the closed region of wounds, as evaluated by CD31, VE-cadherin and fibronectin staining and of activated fibroblasts assessed by vimentin, col1A and FAP1α. These results encourage defining the association mesoglycan/VEGF to activate endothelial and fibroblast cell components in skin wound healing promoting the creation of new vessels and the deposition of granulation tissue.

## Introduction

Neovascularization or angiogenesis is a crucial event in tissue regeneration, including skin wound healing. This process begins immediately after a cutaneous injury and it appears correlated to dynamic and orchestrated phases aimed to realize an efficient and functional re-epithelialization. These phases evolve through a complex of cellular, physiologic, and biochemical events, such as inflammation, granulation, re-epithelialization, and remodeling process. Among these overlapping stages, angiogenesis involves the growth of new capillaries to form granulation tissue starting three to 5 days after tissue injury^1,2^. The new vessels secrete paracrine factors to promote survival of adjacent cells and the recruitment of further cell elements. In particular, when new capillaries become visible in the wound bed, it appeared also favored the formation of granulation tissue, which, in turn, acts as a matrix for proliferating blood vessels, migrating fibroblasts and new collagen deposition^3,4^. In general, during the healing of adult skin wounds, the number of capillaries increases dramatically to a higher level than that in normal tissue^5^. In chronic wounds, angiogenesis appears impaired leading to further tissue harm due to chronic hypoxia and damaged micronutrient delivery. This phenomenon entails the loss of numerous molecules as cytokines, chemokines, and growth factors whose correct gradient is essential for the wound closure^6^. The growth factors, in particular, represent important mediators for each step occurring for tissue regeneration and are, all together, entangled in a complex network of cell recruitment and activation of specific pathways, including angiogenesis itself^7,8^. Among them, it is reported in depth the abnormal low levels of the vascular endothelial growth factor (VEGF) in chronic lesions^9^. VEGF is a homodimeric glycoprotein existing in five isoforms from alternative splicing of its mRNA; among these ones the VEGF165 isoform is the most studied. Its transcription and secretion are elevated in the acute lesions, acting as an endothelial cell mitogen, chemotactic agent, and inducer of vascular permeability^10^. In the process of wound repair, VEGF acts on angiogenesis and tissue granulation at the early stage of healing. Thus, in chronic lesions, characterized by the loss of VEGF, it can represent a possible therapeutic modality if externally administered^11,12^.

Recent studies have proved the promoting effects of mesoglycan in the tissue regeneration. This latter is a natural mixture of glycosaminoglycans, unbranched polysaccharides with repetitive disaccharide units; they are structurally heterogeneous in terms of their carbohydrates composition, chain length, sulfation pattern and degree. It is a preparation extracted from porcine intestinal mucosa and is composed of heparan sulfate (47.5%), dermatan sulfate (35.5%), slow-moving heparin (8.5%) and chondroitin sulfate (8.5%). It is usually commercialized as fibrinolytic drug^13,14^ and has revealed able to act as pro-resolving molecule inducing keratinocytes differentiation and migration, the deposition of granulation tissue, through fibroblasts activation. Moreover, mesoglycan is able to promote the new blood vessels formation and an anti-inflammatory response^15–18^. These effects have allowed considering mesoglycan an appealing therapeutic agent in the treatment of skin wound healing since it has been shown able to act in the main phases of this process, from the injury to the remodeling^19–21^. Indeed, its effects appeared significantly amplified when used in combination with other elements such as the protein lactoferrin. Actually, these two substances help each other notably enhancing the pro-healing and antimicrobial effects^21^.

Our recent work in addition, has highlighted a closed connection between mesoglycan and VEGF in tissue regeneration, describing, for the first time, a combined effect of two molecules that initially have been considered independent pro-angiogenic factors^22^.

Furthermore, it is known that the hypoxic condition characterizing lesions could lead to a slowing of healing processes preventing the pro-repair activity of treatments^23^. Thus, the role of mesoglycan could be enhanced through the combination with a growth factor which specifically could act on vascularization in order to improve oxygen and nutrients contribution. For that reason, we have focused on VEGF whose low levels in chronic lesions need to be supplied by an exogenous administration^9^. On the other hand, the use of VEGF alone could be not enough in the stimulation of the whole range of cells and biological processes necessary for a good re-epithelialization. Actually, this work is aimed to add a new appealing tile in this therapeutic context assessing the promising pro-healing effects of VEGF in combination with mesoglycan. In particular, we have investigated the direct effect of this combination on endothelial cells and its indirect action on fibroblasts. Moreover, the more rapid and structured closure of mice skin wounds in presence of VEGF together with mesoglycan, compared to each substance taken alone, further confirmed the desired positive pro-healing activity.

## Results

### The association of mesoglycan and VEGF promotes in vitro angiogenesis and the recruitment of fibroblasts

**Figure 1:**
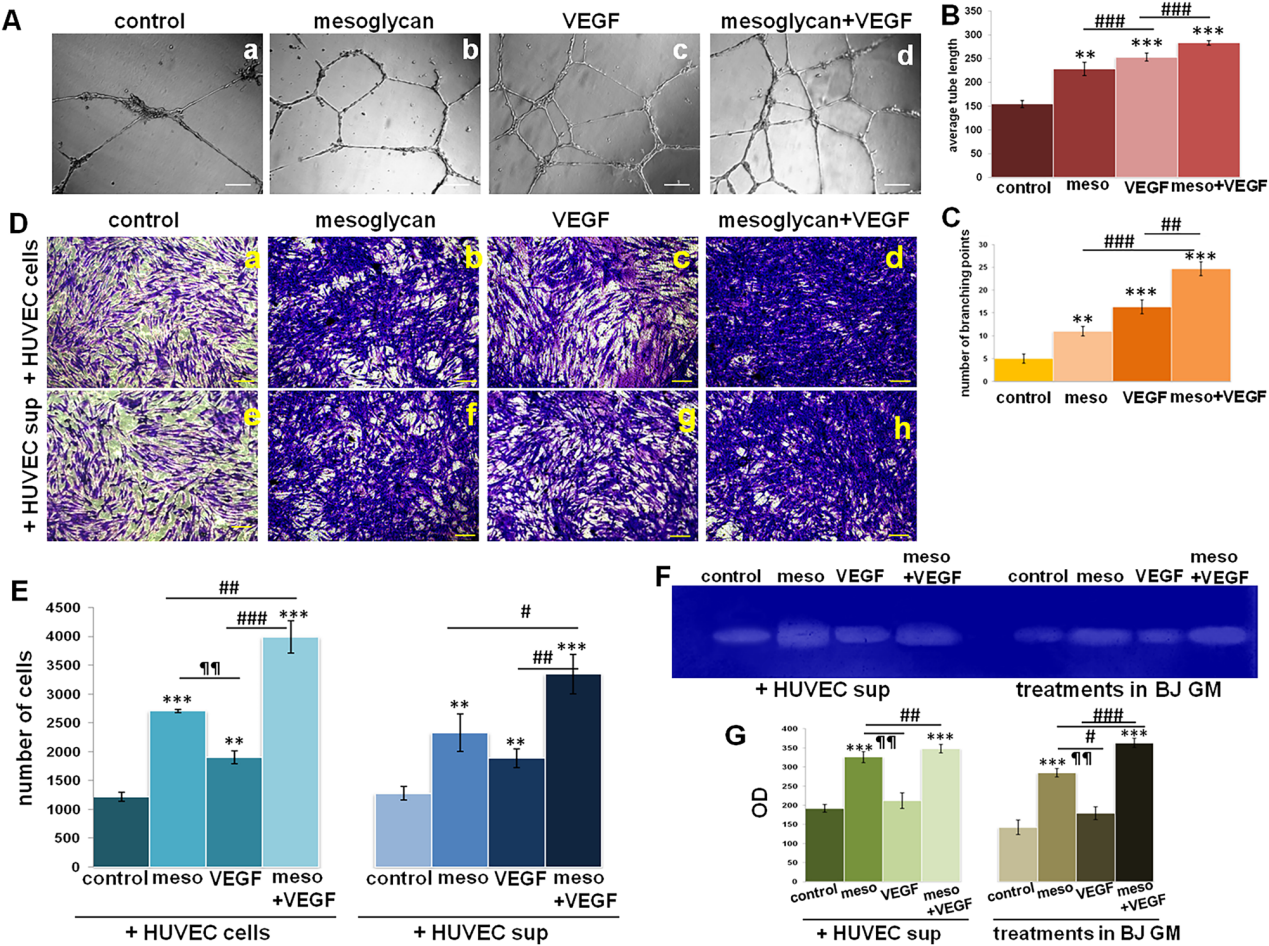
(**A**) Representative bright field images of in vitro angiogenesis (panels a–d) and analysis of (**B**) tube length carried out the Openlab software and (**C**) the number of branches calculated by ImageJ (Angiogenesis Analyzer tool) software on HUVEC cells treated with mesoglycan (meso; 150 μg/ml) and VEGF (10 ng/ml) alone and together. Magnification ×10. Bar = 100 µm. Results of the invasion assay on BJ cells in presence, in the lower chamber, of HUVEC cells pretreated for 24 h with mesoglycan (meso; 150 μg/ml) and VEGF (10 ng/ml) alone and together (panels a–d) and of supernatants harvested from HUVEC cells pretreated for 24 h mesoglycan (meso; 150 μg/ml) and VEGF (10 ng/ml) alone and together (panels e–h). Both (**D**) representative images and (**E**) the analysis of the number of cells have been reported. Magnification ×20. Bar = 150 μm. (**F**) Gelatin zymography showing increased gelatinolytic activity of MMP-2 of BJ cells. (**G**) Densitometry analysis of the intensity of lanes calculated respect the sample volume which was the same for every experimental point. All the data represent a mean of n = 3 independent experiments ± SD based on one-way ANOVA and a two-tailed Student's *t*-test as appropriated, *p < 0.05, **p < 0.01, ***p < 0.001 versus untreated control; ^#^p < 0.05; ^##^p < 0.01; ^###^p < 0.001 versus mesoglycan or VEGF single treatment; ¶¶p < 0.01 for VEGF treatment versus mesoglycan one.

In this work we have used mesoglycan and VEGF as single treatment, but we have mainly focused on the assessment of the effects derived from their combination. First, we have evaluated that the association mesoglycan/VEGF induced a strong *in vitr*o angiogenesis on HUVEC cells, significantly more than each single molecule. This result has been highlighted by representative bright filed images (Fig. 1A, panels a–d) and the analysis of the number of branching points (Fig. 1B) and the average of tube length (Fig. 1C).

The necessary interconnection among cell populations which take action in the different phases of wound repair has led us to study the effects of endothelial cells on fibroblasts. In Fig. 1D,E, we report the invasion ability of BJ fibroblasts in response to chemo-attractive action of HUVEC treated with mesoglycan, VEGF and both of them. The combination of our interest notably promoted fibroblasts invasiveness if compared to single treatments and mainly to not treated cells, as shown by representative cell images and in the histogram reporting the analysis of cell number. Moreover, while following the same trend, BJ have been recruited more rapidly in presence of HUVEC cells in the lower chambers of trans-wells (Fig. 1D, panels a–d and E for the histogram) than in response to the supernatants harvested from HUVEC cells previously treated for 24 h with mesoglycan, VEGF and their association (Fig. 1D, panels e–h and E for the histogram). Moreover, fibroblasts invasion ability has been evaluated also by zymography showing the activation of metalloproteinase 2 (MMP2). In this case, BJ secreted the activated form of the enzyme in a very significant manner when the two molecules have been added together. This data is evident after the direct administration of mesoglycan, VEGF and their combination and also when cells have been treated with HUVEC cells supernatants obtained after a pre-treatment with the single molecules and their mixture (Fig. 1F for gel image and Fig. 1G for the densitometry analysis of the lanes).

### The association of mesoglycan and VEGF stimulates skin wound repair in vivo

**Figure 2:**
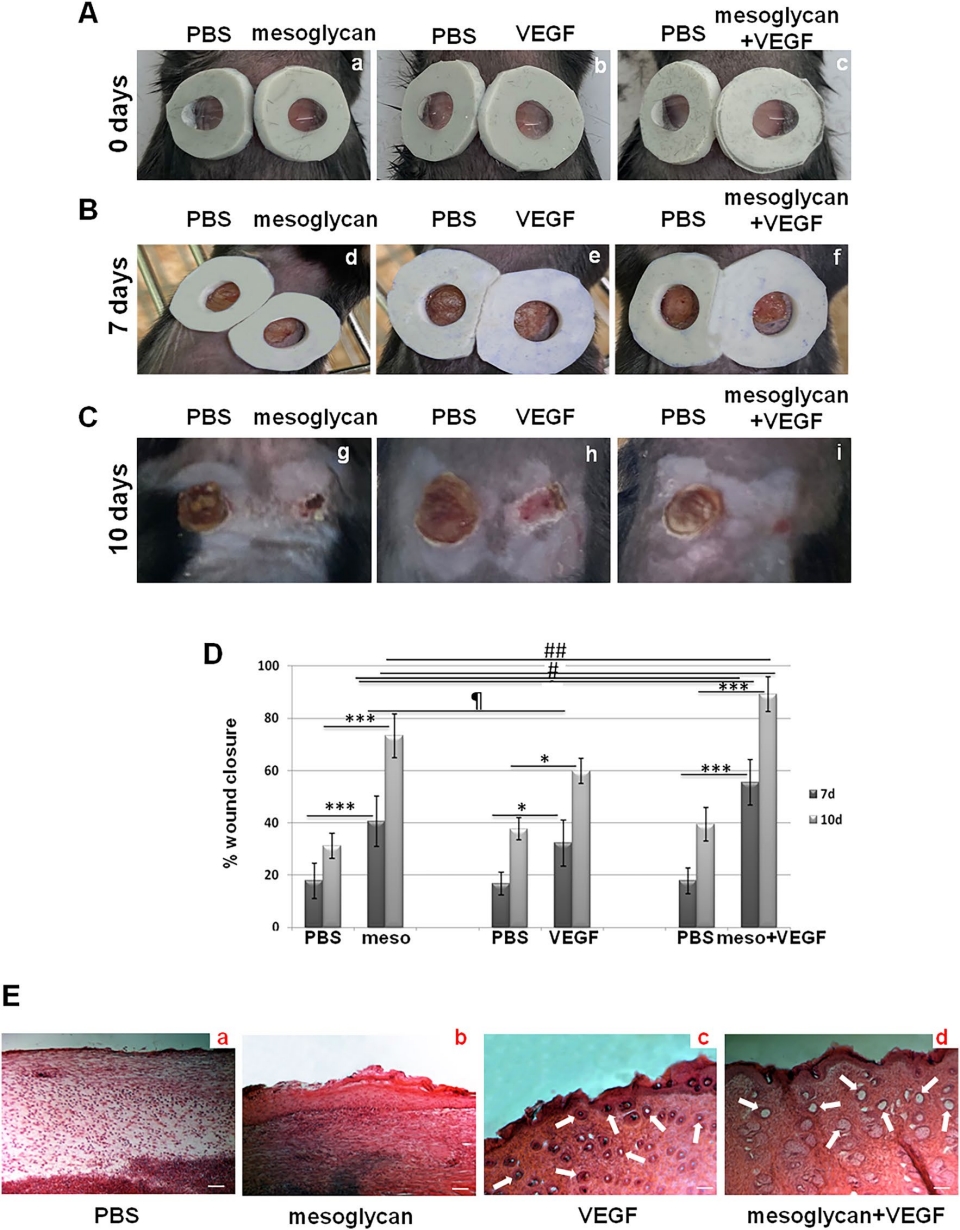
Excisional skin wounds on C57BL6 mice were macroscopically photographed at the indicated time (**A**) the day 0 pictures have been taken immediately after injury, panels a–c; (**B**) the day 7 of treatments has been represented by panels d–f; (**C**) the day 10 refers to the sacrifice, panels g–i. Representative results from five individual mice from each group are shown. (**D**) Wound areas measurements at day 10 calculated as percentage as differences with the diameter of the related lesions at day 0. The data represent a mean of n = 3 independent experiments ± SD based on one-way ANOVA and a two-tailed Student's *t*-test as appropriated; *p < 0.05, ***p < 0.001 versus PBS treated controls; ^#^p < 0.05; ^##^p < 0.01 for combination versus mesoglycan or VEGF treated wounds; ¶p < 0.05 for VEGF treatment versus mesoglycan one. (**E**) Skin sections harvested at day 10 have been stained through H&E (panels a–d). White arrows indicate new blood vessels. Magnification ×10. Bar = 100 µm.

Following the evaluation of the in vitro effects of mesoglycan and VEGF alone and in association, we have continued our study testing them on C57BL/6 mice on which we have created two interscapular skin wounds. The wound areas, easily accessed on the right to apply topical drops of mesoglycan or VEGF or mesoglycan/VEGF, and of PBS on the left, have been recorded by photography for 10 days. First, we confirmed the pro-healing effect of mesoglycan, which is more effective than VEGF, compared to PBS. Interestingly, the association induced the reduction of wound regions with a significant relevance with respect to all the other treatments. This faster wound healing response has been highlighted in the macroscopic photograph reportage (Fig. 2A, panels a–c for the beginning of the experimental phase, Fig. 2B panels d–f at 7 days of treatments and Fig. 2C, panels g–i for the day of sacrifice) and in the analysis of wound sizes (Fig. 2D). Moreover, the H&E staining of mice skin sections, harvested post sacrifice at day 10, revealed a generally unstructured matrix in absence of treatments, characterized by a thinned and slightly dense staining (Fig. 2E, panel a). differently from a compact matrix whose formation has been induced by mesoglycan (Fig. 2E, panel b). A similar structured condition has been found in presence of VEGF accompanied, however, by a relevant number of vessels (Fig. 2C, panel c). The compactness of matrix, as revealed by a dense H&E signal, may allow cell organization and activation to form vessels, by creating a scaffold to guarantee cell motility. Finally, the use of the combination mesoglycan/VEGF induced the formation of a very well organized matrix and of a more important number of vessels (Fig. 2E, panel d) compared to each single treatment.

### The activation of endothelial cells in mice skin lesions is promoted by the association mesoglycan and VEGF

**Figure 3:**
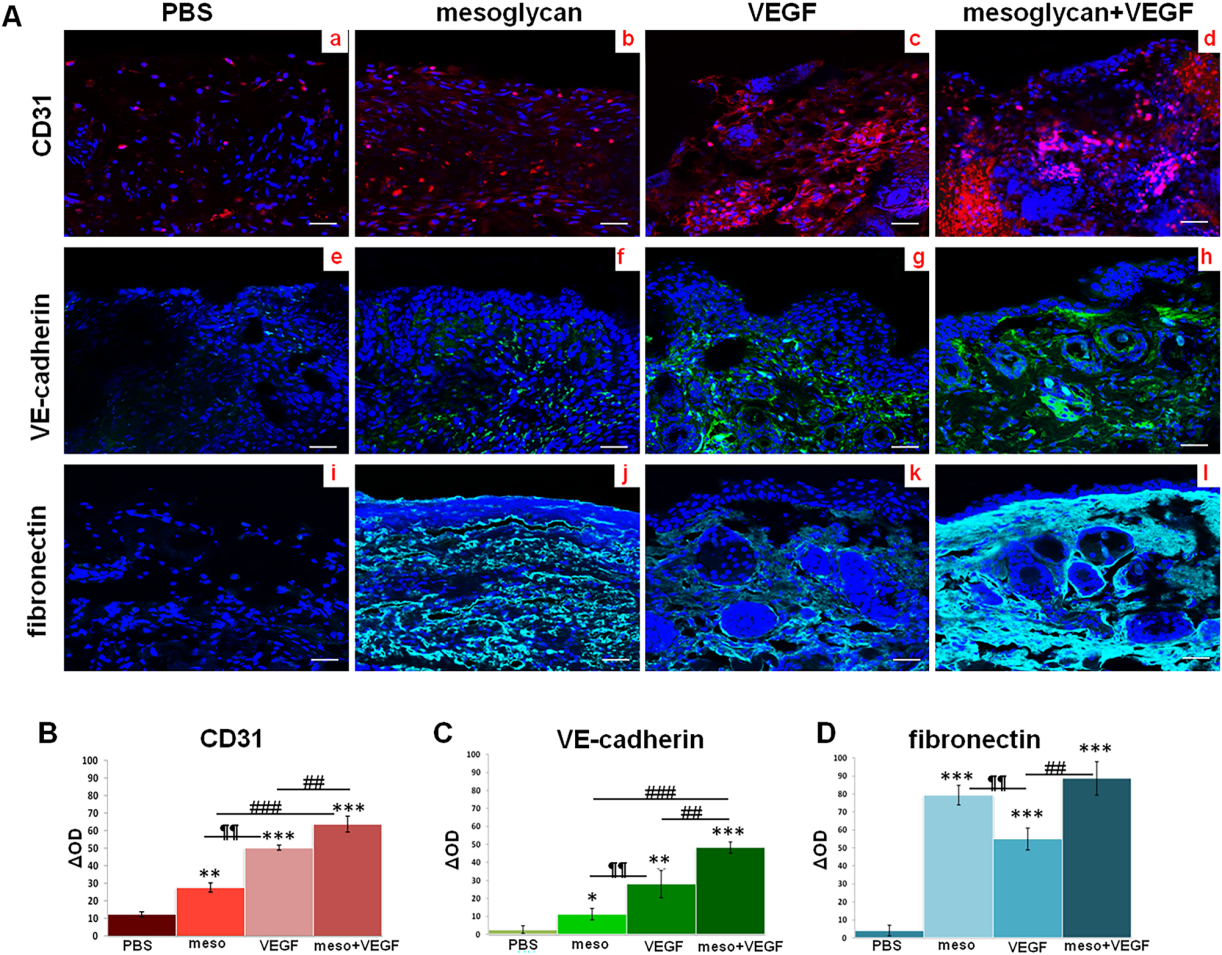
(**A**) Immunofluorescence analysis of mice sections for CD31 (panels a–d); VE-cadherin (panels e–h); fibronectin (panels i–I). Densitometry analysis of fluorescence intensity of the same protein markers (**B**) for CD31; (**C**) for VE-cadherin; (**D**) for fibronectin). The data represent a mean of n = 3 independent experiments ± SD, based on one-way ANOVA and a two-tailed Student's *t*-test as appropriated, *p < 0.05, **p < 0.01, ***p < 0.001 versus untreated control; ^#^p < 0.05, ^##^p < 0.01, ^###^p < 0.001 for combination versus mesoglycan or VEGF treated cells; ¶¶p < 0.01; ¶¶¶p < 0.001 for VEGF treatment versus mesoglycan one.

The appealing data shown above have led us to investigate the activation of endothelial cells and, then, fibroblasts, in the mice injured sites. Thus, we first verified the presence of endothelial cells in the skin biopsies using the endothelial specific marker CD31. The immunofluorescence staining appeared strongly improved after the VEGF treatment much more than the mesoglycan one, compared to the PBS. However, CD31 signal notably increased when the growth factor has been associated to the mixture of glycosaminoglycans (Fig. 3A, panels a–d).

Furthermore, as considerably expressed in endothelial cells, we evaluated the signal of the vascular endothelial (VE)-cadherin protein, showing that following treatments with VEGF, notably more than mesoglycan, its expression increased. In presence of both substances, this protein appeared strongly enhanced (Fig. 3A, panels e–h). Moreover, we tested also the expression of fibronectin in mice sections, revealing that in presence of mesoglycan, more than in case of VEGF treatment, it markedly increased reaching a higher level when the two treatments have been added together (Fig. 3A, panels i–I).

The histograms (Fig. 3B–D) show the densitometry analysis of each marker used for confocal analysis.

### Fibroblasts employment is stimulated in mice skin lesions the association mesoglycan and VEGF

**Figure 4:**
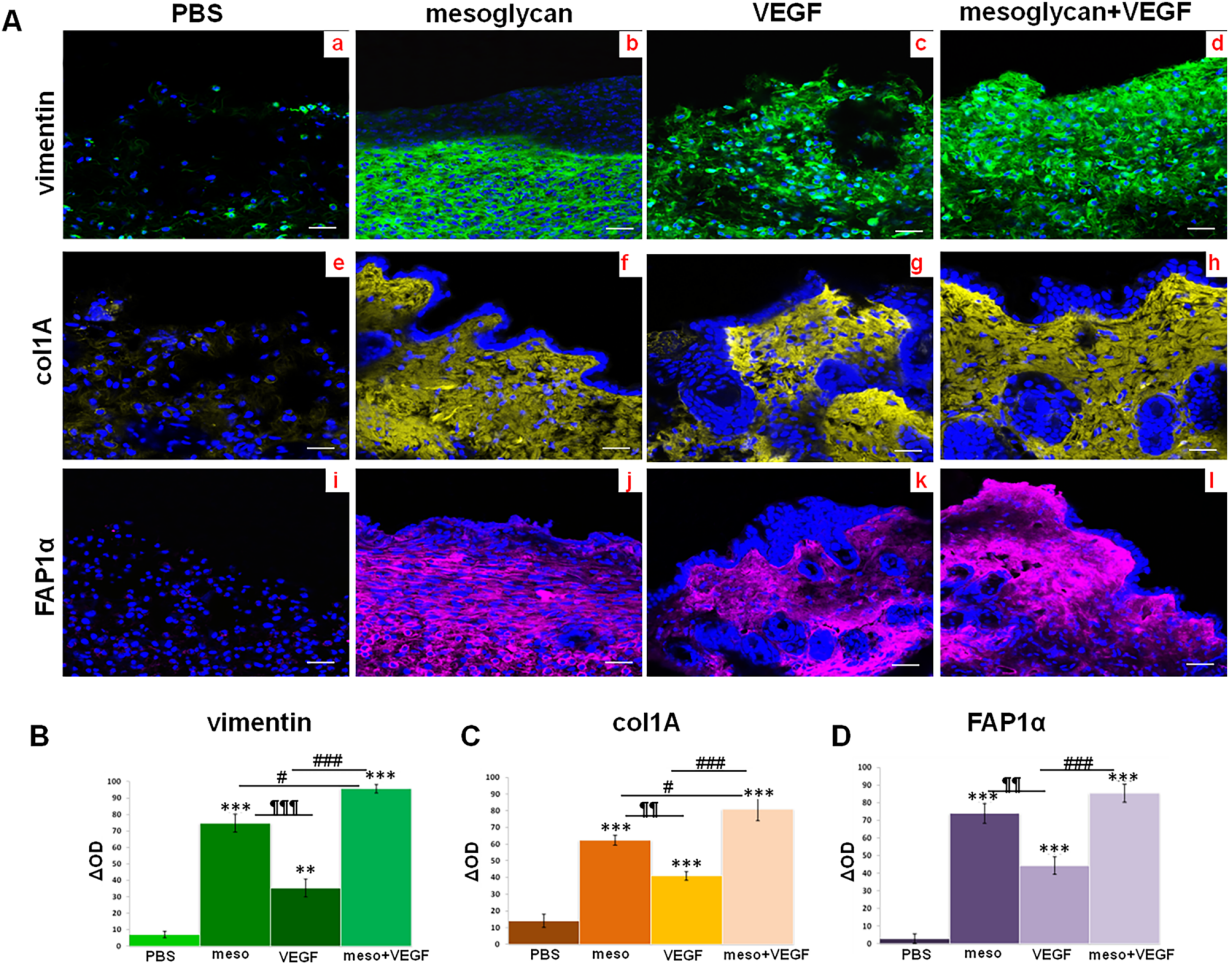
(**A**) Immunofluorescence analysis of mice sections vimentin (panels a–d); col1A (panels e–h); FAP1α (panels i–I). Densitometry analysis of fluorescence intensity of the same protein markers (**B**) for vimentin; (**C**) for col1A; (**D**) for FAP1α). The data represent a mean of n = 3 independent experiments ± SD based on one-way ANOVA and a two-tailed Student's *t*-test as appropriated, *p < 0.05, **p < 0.01, ***p < 0.001 versus untreated control; ^#^p < 0.05, ^##^p < 0.01, ^###^p < 0.001 for combination versus mesoglycan or VEGF treated cells; ¶¶p < 0.01; ¶¶¶p < 0.001 for VEGF treatment versus mesoglycan one.

In order to prove the recruitment of fibroblasts, we used vimentin and assessed that the biopsies treated for 10 days with mesoglycan and VEGF in co-treatment showed a significant high level of this protein. This signal appeared stronger than PBS and also than single treatment. In this case, mesoglycan retained a greater ability to enlist fibroblasts through the lesions compared to VEGF (Fig. 4A, panels a–d).

Next, the tracking of collagen type I A further revealed the fibroblasts recruitment to wounds and above all the ability of these cells to deposit an organized extracellular matrix during tissue regeneration. The most important impact on this activity has been obtained in presence of mesoglycan/VEGF compared to PBS and the single kind of substances (Fig. 4A, panels e–h).

Finally, we showed the presence of fibroblast activated protein (FAP) 1α. Therefore, in mice skin wounds treated with mesoglycan and with VEGF separately the level expression of this protein were significant higher compared with the PBS treated wounds. This data acquires much more relevance when the two treatments are performed together (Fig. 4A, panel i–I).

The histograms (Fig. 4B–D) show the densitometry analysis of each marker used for confocal analysis which clearly shows the significant different expression following the treatments.

## Discussion

The most used dressings for the treatment of skin ulcers must have some specific features to stimulate the healing cascade and speed up the correct regeneration. These characteristics include the maintenance of a moist environment, the enhancement of the epidermal migration, angiogenesis and connective tissue synthesis. Gas exchanges between wound tissue and environment have to be allowed together with an appropriate tissue temperature to improve the blood flow and control the exudates. Furthermore, these devices have to provide protection against bacterial infection, should be non-adherent to the wound and easy to remove after healing^24^. New types of topical dressings based on “active” molecules, as the growth factors, and/or on cell therapy to accelerate closure of non-healing wounds, have contributed to radically transform the field of chronic skin ulcers management^25,26^.

Our in vitro data have revealed the strong activation of endothelial cells by the association mesoglycan/VEGF and proved the pro-invasive effects of these cells on fibroblasts. These findings suggest the importance of the extra-angiogenic effects of VEGF on wound repair which recently have been established^9^. Indeed, we have chosen to associate mesoglycan with VEGF since in case of hypoxic condition, the main cell populations could be not sufficiently able to answer to the activation process favored by the mixture of glycosaminoglycans. Thus, by the formation of new vessels by VEGF, these processes could be interestingly promoted. This concept has been further highlighted by the in vivo findings of the acceleration of repair mice lesions by mesoglycan/VEGF, much more than each single treatment. Next, the confocal analysis has confirmed the presence of endothelial cells in the site of injury through the CD31 and VE-cadherin and fibronectin. The strong engagement of this last protein, closely correlated to the autocrine regulation of vascular morphogenesis^27,28^, demonstrates that the mesoglycan/VEGF combination promotes the deposition of ECM proteins. In this way, it directly contributes to the formation of a scaffold for cell migration, already considered essential for the correct dermal homeostasis^29^. However, the most interesting aspect suggested by this result focuses on the ability of fibronectin to bind growth factors, including VEGF, and to define a real gradient of these chemoattractive molecules accentuating their regenerative effects^30^. Similarly to what we have assessed about fibronectin, the increased expression of col1A induced by the association supplies another interesting aspect for the maintenance of the appropriate environment to start the remodeling phase of the wound healing. The activation of fibroblasts in vivo, further proved by the vimentin and FAP1α signals, is well known to promote the granulation phase as key moment of skin wound healing. It is not by chance that collagen-based matrices facilitate migration of fibroblasts providing a solid structural scaffold. This latter encourages new tissue regeneration, protects proteins involved in the wound healing process and stimulates angiogenesis through engagement of specific integrin receptors^31,32^. Thus, the increase of fibronectin and col1A expression at the injury site has interestingly suggested the creation of a positive loop for which fibroblasts, activated by endothelial cells, endorse the formation of new vessels by the deposition of ECM as scaffold and by the secretion of other growth factors amplifying, in their turn, the endothelial cells activation. This potential loop represents an attractive issue for which the use of mesoglycan/VEGF can indirectly promote the deposition of this macromolecule instead of bringing it in from outside by a specific dressing. Indeed, the matrix-based biomaterials or similar compositions require additional devices to intervene in the different phases of tissue regeneration and separately activate the cell elements and molecules involved.

Taken together, these data are in agreement with the knowledge of VEGF functions in the induction of wound healing flow, including angiogenesis, epithelialization, and collagen deposition^10,33^. The secretion of VEGF by fibroblasts under specific stimuli as hypoxia in wound healing has been described in depth as aimed for the angiogenesis promotion^34^. However, the effects on fibroblasts directly mediated by VEGF have been intriguing found as able to induce the production of fibronectin, as also suggested in this work^35^. Furthermore, our results are interestingly overlapped with our previous finding for which VEGF and mesoglycan are two components which, in concert, retain notable pro-angiogenic effects. In particular, this novel mechanism requires the interaction of mesoglycan on the co-receptors syndecan 4 on endothelial cells which are activated and secrete microvesicles. These latter are able to promote, in an autocrine manner, the externalization of VEGF-A, which in turn stimulates the VEGF-receptor 2 (VEGFR2) further supporting the formation of new capillary-like structures^22^. Mesoglycan has been shown also able to markedly promote fibroblasts activation in injury sites of patients affected by pressure ulcers^19^. Moreover, many evidences highlight that these cells retain an important angiogenic action triggering the neovessel formation through several different mechanisms. These mechanisms include secretion of cytokines, chemokines, and growth factors, and secretion of ECM proteins with angiogenic properties^36^. The consideration for which this intricate cross-talk can be induced by the combination of mesoglycan and VEGF makes this one an appealing novel therapy strategy in the contest of skin chronic ulcers. Indeed, the endothelial cells and fibroblasts are usually linked in homeostasis and their communication has been at the base of different kinds of coculture systems as new tissue engineering strategies to promote vascularization^36,37^. For that reason, our findings strongly highlight the potential usefulness of modulating these effects for therapeutic purposes, in particular through the creation of a synergistic activity in the induction of multiple effects.

Insufficient wound vascularization, stemming from the low activity of growth factors, such as VEGF, likely contributes to compromising delays in the repair process. Thus, it is still necessary to investigate about the development of new topical devices able to function as inducer of cell activation in the dynamic multilateral promotion of wound repair. Additionally, our in vivo procedure based on the use of wild-type mice only contributed to the investigation about the repair of an acute injury. Indeed, these latter are known to usually proceed through an organized and appropriate regeneration process sustained by anatomical and functional integrities. Inversely, chronic wounds are a consequence of extrinsic factors as age, vascular, metabolic and autoimmune diseases, and undergo a delayed or unsuccessful healing process^38^. Thus, the winning pharmacological approach used to correctly accelerate an acute injury could not preserve the same degree of results in a chronic condition. This important difference represents for us the key of an important challenge aimed to the finding of advanced systems able to promote the formation of a functional structure characterized by an anatomical continuity also in presence of all those elements causing chronic wounds.

**Figure 5:**
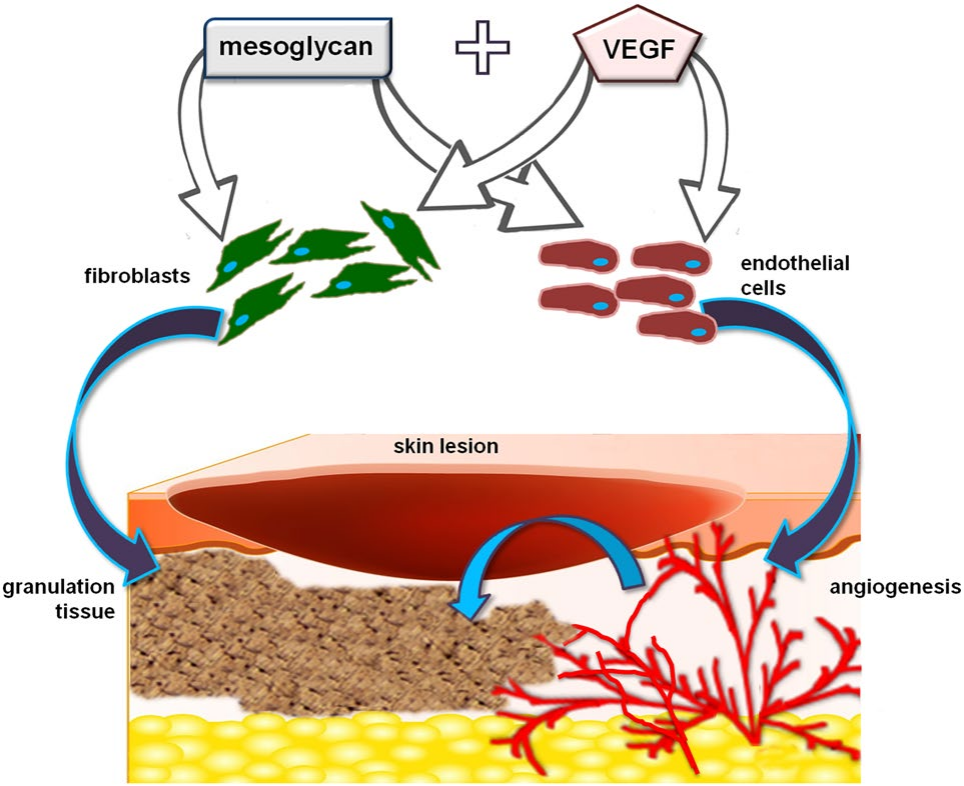
Mesoglycan and VEGF, both of them, directly trigger the activation of fibroblasts and endothelial cells. Thus, in wound repair, granulation and angiogenesis processes are induced. Moreover, endothelial cells, once engaged in the formation of the new vessels, further promote the deposition of granulation tissue by fibroblasts. In this way, a positive feed-back is induced by the combination of mesoglycan and VEGF favoring the cross-talk between two of the crucial cell populations involved in tissue regeneration.

In this scenario and based of these promising data, this study has provided new insights about the pro-angiogenic activity of mesoglycan supported by VEGF. These molecules, together, have appeared further able to recruit and activate fibroblasts as target, in turn, of endothelial cells. In this way, by the use of a single device the complex objective of wound repair can be reached through a multifaceted approach. This consideration encourages the evaluation of a new kind of topical device releasing mesoglycan and VEGF whose association significantly promotes the close interconnection between endothelial cells and fibroblasts. They can therefore represent an attractive winning therapeutic approach in tissue regeneration (Fig. 5). Our future efforts will be based on the investigation of other types of cell–cell or cell-ECM cross-talk, in order to have a complete knowledge of the various pieces of the possible mechanism of action of the combination.

## Methods

### Cell cultures and preparation of mesoglycan and VEGF

HUVEC cell line (Human umbilical vein endothelial cells) (American Type Culture Collection ATCC PCS-100-010™; Manassas, VA USA) were cultured as reported in^20^. Briefly, it was maintained in endothelial growth medium (EGM-2) medium contains EBM-2 medium (serum free, growth-factor free), supplemented with 2% fetal bovine serum (FBS), human fibroblast growth factor-B (hFGF-B), human epidermal growth factor (hEGF), human vascular endothelial cell growth factor (hVEGF), long R insulin-like growth factor-1 (R3-IGF-1), ascorbic acid, hydrocortisone, and heparin (Lonza; Basel, Switzerland). BJ cell line (Human immortalized fibroblast) were purchased from ATCC (Manassas, VA USA; CRL2522™) and cultured in Eagle's Minimum Essential Medium (MEM) with 10% FBS, 1% l-glutamine, 1% Sodium Pyruvate, 1% NEAA. Cells were stained at 37 °C in 5% CO_2_-95% air humidified atmosphere and were serially passed at 70–80% confluence.

The composition of sodium mesoglycan is reported in^13^, it was provided by LDO (Laboratori Derivati Organici spa; Trino, VC, Italy) and dissolved in cell medium or PBS. It was used at a final concentration of 150 µg/ml as reported in^21^ both alone and in association. VEGF (Recombinant Human VEGF 165 Protein; R&D Systems; Minneapolis, MN, USA) was dissolved in PBS reaching a final concentration of 10 ng/ml as described in^39,40^ in case of single administration and with mesoglycan.

### Invasion assays

BJ invasiveness was studied using the trans-well cell culture (12 mm diameter, 8.0-fim pore size; Corning Incorporated; NY, USA), as previously described^41^. In particular, the upper front of trans-well cell culture (12 mm diameter, 8.0-fim pore size; Corning Incorporated; NY, USA) was coated with matrigel (Becton Dickinson Labware; Franklin Lakes, NJ, USA), diluted with 3 volumes of serum-free medium and stored at 37 °C until its gelation. Cells were plated in 350 μl of serum-free medium at a number of 5 × 10^4^/insert in the upper chamber of the trans-well coated with matrigel diluted with 3 volumes of serum-free medium. In the lower chamber, it had been plated HUVEC cells pretreated 24 h before with mesoglycan 150 µg/ml in association or not with VEGF 10 ng/ml. Other experimental points have been performed using in the lower chamber the supernatant of HUVEC cells treated 24 h before as reported above. The schematic representation of co-culture system is reported in Supplementary information. After 24 h, as reported in^42^, the medium was aspirated, the filters were washed twice with PBS 1× and fixed with 4% p-formaldehyde for 10 min, then with 100% methanol for 20 min. The filters so fixed, were stained with 0.5% crystal violet prepared from stock crystal violet (powder, Merck Chemicals, Darmstadt, Germany) by distilled water and 20% methanol for 15 min. After that, the filters were washed again in PBS 1× and cleaned with a cotton bud.

### Tube formation assay

As reported in^43^, a 24-well plate was coated with matrigel (Becton Dickinson Labware, Franklin Lakes, NJ, USA) mixed to EGM-2 1:1 on ice and incubated at 37 °C for 30 min to allow gelation to occur. HUVEC cells were added to the top of the gelated matrigel at a density of 2 × 10^4^ cells/well in the presence or absence of the treatments indicated for each experimental point. After 12 h, the images were captured using the EVOS optical microscope (10×) (Life technologies Corporation; Carlsbad, CA, USA). The images were imported as TIFF files into the Openlab software (Improvision Inc) program as reported in^44,45^. Tube formation was quantified by measuring the long axis of each tube in 10 random fields per well at low power (10×) magnification by using Openlab software. The mean tube length was determined for each well. The number of the branching points has been calculated through the ImageJ software (NIH, Bethesda, MD, USA) (Angiogenesis Analyzer for ImageJ).”

### Gelatin gel zymography

Gelatinolytic activity was detected by SDS-PAGE zymography, as reported in^42^. Briefly, serum-free supernatant samples were analyzed under non-reducing conditions without boiling, through a 10% SDS–polyacrylamide gel co-polymerized in the presence of gelatin 0,1% (Sigma-Aldrich; St. Louis, MA, USA). After the electrophoresis run, carried out at 125 V, the proteins in the gel were renatured in a 2.5% Triton X-100 solution for 1 h. The gel was then incubated with 50 mM Tris–HCl, pH 7.8, 200 mM NaCl, 5 mM CaCl_2_ and 5 μM ZnCl_2_ at 37 °C for 48 h, which allows substrate degradation. Finally, the gels were stained with 0.5% Coomassie Brillant Blue R-250 (BIO-RAD, Hercules, CA, USA). Proteolytic bands were visualized by destaining with 10% methanol and 5% acetic acid. The areas of digestion appeared as light bands on a dark stained background where the substrate was degraded by the enzyme. The volume of each sample has been loaded in the same amount and the optical density (OD) of each band has been analyzed through the ImageJ software (NIH, Bethesda, MD, USA).

### In vivo wound modeling and analysis

C57BL/6 mice (6–8 week-old females) were obtained from Charles River (Italy) and bred under pathogen-free conditions in the Animal Facility of the University of Salerno. Animal experiments were approved by University of Salerno Ethic committee and the Italian Health Ministry (authorization n. 489/2018-PR) in compliance with international laws and policies (EU Directive 2010/63/EU and Italian D.Lgs 26/2014). The study was carried out in compliance with the ARRIVE guidelines. The detailed procedure about the in vivo experiments and the analysis post-sacrifice has been described in^21^. All methods were carried out in accordance with relevant guidelines and regulations. A total of 10 animals per experimental group have been used and all experimental procedures were done in a hood under sterile condition maintained throughout. Full-thickness wounds were made in the doughnut region of the splint using 6-mm-diameter punches (Acuderm, Fort Lauderdale, FL, USA). Around the wounds a sterile silicone sheet ring has been carefully placed previously treated with an instant-bonding adhesive (super attak, Loctite^®^; Henkel, Milan, Italy) so that the wound is centred within this created splint. The use of these rings, as reported in^46^, have prevented wound closure caused by skin contraction and thus allowing wounds to heal through granulation and re-epithelialization similar to that in humans. In our case, the silicone rings have also prevented that the solutions touch each other. Treatments have been performed using PBS as control. Mice have been treated with 50 µl of PBS solution containing or not mesoglycan (150 µg/ml as final concentration) and VEGF (10 ng/ml) every 2 days from day 0 to day 10 and a photography reportage has been obtained at days 0, 3, 7 and 10. The percentage of the initial wound that remained open was quantified at different time points (days 0, 7, and 10). After the mice were sacrificed, at day 10 the skin wounds were harvested at about 50 mm on the side of lesions including both of them for each sample biopsy, washed and fixed in a solution of p-formaldehyde. Then biopsies were incubated in a sucrose solution to guarantee the cryoprotection. The obtained skin biopsies were cut on a Leica CM 1950 cryostat at 10–12 μm, mounted directly on super frost slides (Thermo Scientific; Waltham, MA, USA), and processed for haematoxylin and eosin (H&E) staining as reported in^47^. Briefly, cryostat sections were dehydrated for 5 min with cold acetone and then rehydrated. Next, slides were placed in haematoxylin stain for 9 min, rinsed in alcoholic acid, differentiated in 80% alcohol and stained with eosin for 2.5 min, rinsed in 95% ethanol, dehydrated with absolute ethanol and cleared in xylenes for 4 min. The images were taken through the Axio Observer microscope (4, 10 and 40×) (Carl Zeiss MicroImaging GmbH; Jena, Germany).

About the immunofluorescence assay, mice sections have been incubated O/N with anti-CD31 (1:100; Abcam; Cambridge, UK); anti-VE-cadherin (1:100; Santa Cruz Biotechnologies; Dallas, TX, USA); anti-fibronectin (1:100; Santa Cruz Biotechnologies; Dallas, TX, USA), anti-vimentin (1:250; Santa Cruz Biotechnologies; Dallas, TX, USA); anti-col1A (1:250; Santa Cruz Biotechnologies; Dallas, TX, USA); anti-FAP1α (1:100; Santa Cruz Biotechnologies; Dallas, TX, USA) as described in^20,43^. The staining with conjugated anti-mouse and anti-rabbit antibodies and the nuclei and the confocal analysis were performed as previously described^48^. For immunofluorescence analysis and quantification, the final images were generated using Adobe Photoshop CS4, version 11.0. Quantifications were performed from multichannel images obtained using a 40× objective using ImageJ software (NIH, Bethesda, MD, USA). Based on the magnification used, the region of interest has been calculated as able to cover the great part of lesion in each section and the integrated densities per area from the appropriate channel has been assessed. A minimum of 10 areas were analyzed for each data set, in particular each treated point has been compared to the control of the same section in order to enlarge the statistical evaluation above all about the PBS treated section. The obtained mean value was used to compare experimental groups.

### Statistical analysis

Data analyses and statistical evaluations were carried out using Microsoft Excel; the number of independent experiments, standard deviation, and p-values are indicated in the figure legends. All results are the mean ± SD of at least 3 experiments performed in triplicate. Statistical comparisons between two groups or among more groups were made by using Student’s two-tailed t-test and one-way ANOVA, respectively. Differences were considered significant if p < 0.05, p < 0.01 and p < 0.001.

## Supplementary Information

Supplementary Figure 1.

## Data Availability

The datasets used and/or analyzed during the current study available from the corresponding author on reasonable request.
