# Supplementary figures and images for: The combination of mesoglycan and VEGF promotes skin wound repair by enhancing the activation of endothelial cells and fibroblasts and their cross-talk
Source: Sci Rep. 2022 Jun 30;12:11041. doi: 10.1038/s41598-022-15227-1 (PMC9247059; doi:10.1038/s41598-022-15227-1)

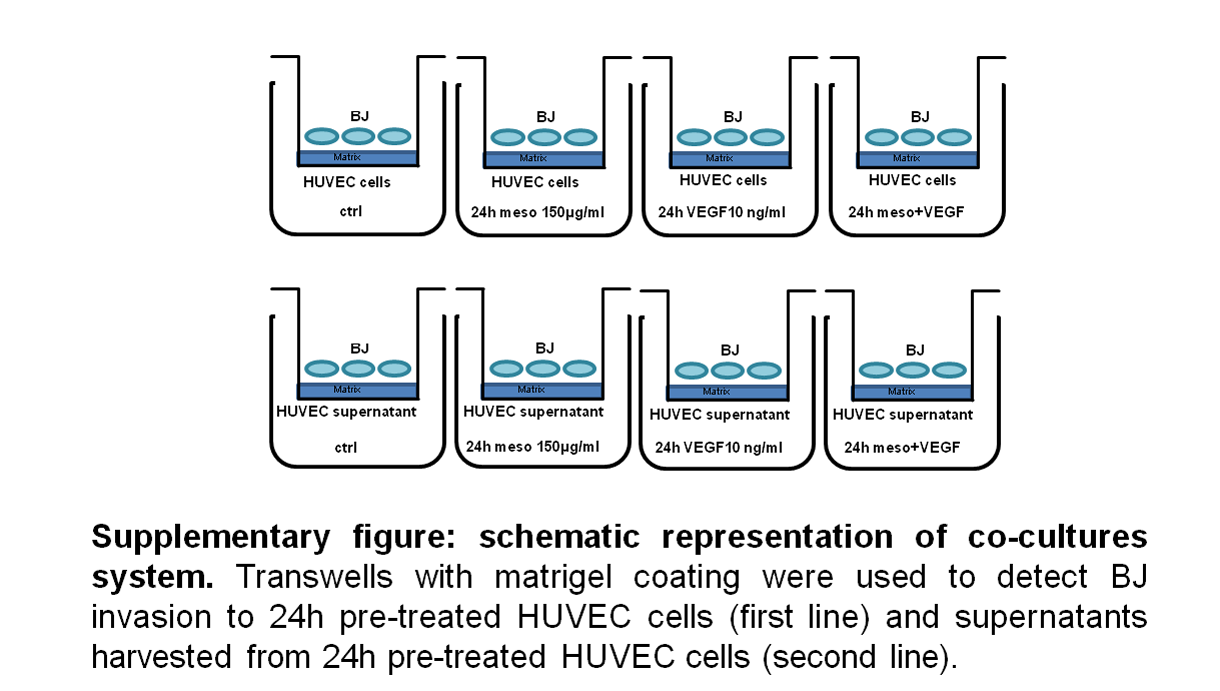

Supplement: Supplementary file 1 — Supplementary Figure 1. [file 41598_2022_15227_MOESM1_ESM.tif]
